# Limited integration of one health and antimicrobial resistance within biodiversity strategies

**DOI:** 10.1186/s12982-025-00974-z

**Published:** 2025-10-14

**Authors:** Carly Ching, Muhammad H. Zaman, Veronika J. Wirtz

**Affiliations:** 1 Department of Biomedical Engineering, Boston University, 44 Cummington Mall, Boston, MA 02215 USA; 2 Center on Forced Displacement, Boston University, Boston, MA USA; 3 Department of Global Health, Boston University School of Public Health, MA 02118 Boston, USA

**Keywords:** Biodiversity, AMR, Antimicrobial resistance, Antibiotic, Policy, One Health

## Abstract

Antimicrobial resistance (AMR) is a One Health policy issue, which connects humans, animals and the environment. Antibiotics and AMR impact ecosystems, yet microbes (which are also part of ecosystems) are often overlooked in biodiversity strategies. This perspective examines the limited integration of AMR-related topics in current biodiversity strategies and discusses barriers and recommendations for inclusion. Documents reviewed include the Kunming-Montreal Global Biodiversity Framework and seven different biodiversity strategies from various regions.

## Introduction

There has been a concerted push by international organizations and experts to approach and frame antimicrobial resistance (AMR) as an interdisciplinary One Health issue (in which human health, animal health and the environment are interconnected domains) [1]. This is supported by both theoretical and demonstrated evidence that illustrate the connections between sectors related to AMR [1]. However, operationalizing One Health strategies has proven to be challenging. A systematic review of challenges in designing and implementing the One Health approach identified several thematic challenges including policy and funding, education and training, surveillance, collaborations and evidence [2]. Moreover, One Health often remains anthropocentric, with unequal value between domains, with the environmental dimension of AMR remaining especially under studied [3].

While some work has studied the implementation of One Health networks and initiatives for AMR, it is less known whether such One Health Networks has led to the effective joining of AMR to relevant independent strategies or policies in practice, or whether silos remain [4–6]. Specifically, related to environmental policy, antibiotic use and AMR has negative consequences on biodiversity, pollution and climate change [1, 7]. Furthermore, biodiversity and ecosystem loss lead to pathogen spillover and increases the risk of human exposure to zoonotic pathogens [8, 9], while climate change can also increase the spread of infectious diseases [10]. As such, policymakers should recognize the importance of integrating AMR into environmental policy, and AMR policymakers should consider the importance of environmental policy. In practice, these connections may be overlooked when working in silos.

Here, we specifically examine the shared interests between antibiotic-reducing activities and biodiversity goals and the limited integration of AMR-related topics in current biodiversity strategies. We further discuss barriers and recommendations. For this work, we define biodiversity strategies as activities and plans to promote the variety of life on earth in general, or the variety of living things in each ecosystem or region, based on the Convention on Biological Diversity definitions [11]. Biodiversity plays a vital role in ecosystem services, which are the services that nature supplies. These include pollination, climate regulation, flood protection, soil fertility and the supply of food, fuel, fiber and medicines [12].

To assess biodiversity strategies and policies for integration of One Health AMR topics, we analyzed selected biodiversity strategies and policies for explicit mention of One Health, antibiotics, antibiotic or antimicrobial resistance and bacteria/microbes/microorganisms. We then read the whole text to identify areas of alignment within each document that AMR or antibiotic use could relate to. Documents included the Kunming-Montreal Global Biodiversity Framework (GBF) (Table 1) and seven different biodiversity strategies listed in Table 2 which had global (and High-income (HI) and Low-and-middle-income (LMIC)) representation.

## The microbial dimension of biodiversity

We chose biodiversity strategies as a case study as there is a direct impact on antibiotic use and AMR to functional ecosystems, which consist of plants, animals, fungi and microorganisms along with the abiotic environment [13, 14]. Specifically, bacteria and fungi are important for ecological functions such as nutrient cycling and decomposition in soil. Antibiotic pollution (i.e., runoff from agricultural practices, hospital and manufacturing waste) can disturb the native microbial community by altering taxa diversity, composition, enzyme activity and structure. This can lead to a loss of biomass and altered microbial activity that includes nitrification, denitrification, and respiration, as well as selection of resistant pathogens [14–17]. However, it should be noted that not all antibiotics have the same effect on biodiversity and evidence remains varied [15, 16], which can be a barrier to integrated policies. This may be because there are many environmental and behavioral factors that impact bacteria and AMR development [18, 19] and shift outcomes. For example, climatic factors such as temperature, rainfall and humidity can also impact AMR development in different ways [15, 20]. Additionally, specific properties of the antibiotic residues, soils and bacteria, as well as methodological differences, can add to the variance of results [15, 16]. Moreover, it is difficult to measure the specific contribution of AMR related to biodiversity to clinical outcomes [21].

Many countries committed to national biodiversity strategies and action plans after adopting the Kunming-Montreal Global Biodiversity Framework (GBF) at the UN Biodiversity Conference (COP15) in December 2022 [22]. This framework includes four goals and 23 targets. Notably, the GBF does not mention microbes (only human, animal and plant groups) but does describe implementation *“with consideration of the One Health Approach*,* among other holistic approaches that are based on science*,* mobilize multiple sectors*,* disciplines and communities to work together*,* and aim to sustainably balance and optimize the health of people*,* animals*,* plants and ecosystems”* [22]. However, as described above, bacteria are an important part of ecosystems. The goals of the GBP relate to sustaining biodiversity and maintaining ecosystem function and integrity. Notably, antibiotic use in animals is a major practice of concern for AMR and sustainable production, and agrifood sectors including livestock production are directly related to more than half the GBP targets, and to all other targets in some way [23]. Specifically, targets related to AMR and antibiotic use are presented in Table 1.

**Table 1:** Targets from Kunming-Montreal global biodiversity framework (GBF) [22]

Target	Interests related to AMR/antibiotic use
TARGET 7 “Reduce pollution risks and the negative impact of pollution from all sources by 2030, to levels that are not harmful to biodiversity and ecosystem functions and services, considering cumulative effects, including: (a) by reducing excess nutrients lost to the environment by at least half, including through more efficient nutrient cycling and use; (b) by reducing the overall risk from pesticides and highly hazardous chemicals by at least half, including through integrated pest management, based on science, taking into account food security and livelihoods; and (c) by preventing, reducing, and working towards eliminating plastic pollution [22].”	Pollution of antibiotics into water and soil can lead to disruption of microbial diversity and AMR which can impact food security and livelihoods [14–16]. • Pollution • Soil Health • Food Security and Livelihoods • Ecosystem Functions
TARGET 10 “Ensure that areas under agriculture, aquaculture, fisheries and forestry are managed sustainably, in particular through the sustainable use of biodiversity, including through a substantial increase of the application of biodiversity friendly practices, such as sustainable intensification, agroecological and other innovative approaches, contributing to the resilience and long-term efficiency and productivity of these production systems, and to food security, conserving and restoring biodiversity and maintaining nature’s contributions to people, including ecosystem functions and services[22].”	Antibiotics are regularly used in agriculture and aquaculture but are not biodiversity friendly. • Sustainable agriculture • Food Security • Ecosystem Functions
TARGET 11 “Restore, maintain and enhance nature’s contributions to people, including ecosystem functions and services, such as the regulation of air, water and climate, soil health, pollination and reduction of disease risk, as well as protection from natural hazards and disasters, through nature-based solutions and/or ecosystem-based approaches for the benefit of all people and nature [22].”	Microbes are an essential part of functioning ecosystem functions and soil health. Antibiotic pollution and AMR disrupt soil health and increase disease risk [17]. • Ecosystem Functions • Soil Health • Disease Risk
TARGET 15 “Take legal, administrative or policy measures to encourage and enable business, and in particular to ensure that large and transnational companies and financial institutions: (a) Regularly monitor, assess, and transparently disclose their risks, dependencies and impacts on biodiversity, including with requirements for all large as well as transnational companies and financial institutions along their operations, supply and value chains, and portfolios; (b) Provide information needed to consumers to promote sustainable consumption patterns; (c) Report on compliance with access and benefit-sharing regulations and measures, as applicable; in order to progressively reduce negative impacts on biodiversity, increase positive impacts, reduce biodiversity-related risks to business and financial institutions, and promote actions to ensure sustainable patterns of production [22].”	Sustainable consumption of meat and organic meat is related to reduction of antibiotic use in livestock [24, 25]. • Sustainable agriculture
TARGET 16 “Ensure that people are encouraged and enabled to make sustainable consumption choices, including by establishing supportive policy, legislative or regulatory frameworks, improving education and access to relevant and accurate information and alternatives, and by 2030, reduce the global footprint of consumption in an equitable manner, including through halving global food waste, significantly reducing overconsumption and substantially reducing waste generation, in order for all people to live well in harmony with Mother Earth [22].”	Sustainable consumption of meat and organic meat is related to reduction of antibiotic use in livestock [24, 25]. • Sustainable agriculture

## One health and AMR is lacking in biodiversity strategies

After analyzing seven different biodiversity strategies from across the globe (Table 2), we found that there was little to no mention of microbes, antibiotics or AMR (3/7 strategies did not have any mentions), nor reference to One Health (2/7 strategies mentioned the term One Health) (Table 2). Notably, however, unlike the GBF, some strategies (4/7) included microbes or bacteria as part of the ecosystem and biodiversity. Like the GBF targets, we identified multiple (and similar) areas of alignment of biodiversity strategies with AMR One Health strategies. Overall, areas for alignment to GBF and current strategies can be largely summarized to: Pollution, Soil Health, Food Security, Ecosystem Functions and Sustainable Agriculture. In short, pollution of antibiotics and runoff from heavy use of antibiotics in agriculture into water and soil can lead to disruption of microbial diversity and ecosystem functions, impact soil function and health, and lead to increased AMR, which can impact food security and livelihoods [14–17]. These areas are largely covered in all the strategies and policies in Table 2, however, with minimal discussion of antibiotics or AMR.

**Table 2:** All explicit mentions of microbes, antibiotics, AMR or one health

	Explicit mentions of microbe, antibiotics or AMR	One Health mentioned
Canada’s 2030 Nature Strategy: Halting and Reversing Biodiversity Loss in Canada* [26]	*“From a One Health perspective—which recognizes the interconnectedness of human*,* animal*,* and environmental health—biodiversity loss will have a significant impact on the health of each component of this system. The current pace of extinctions*,* the encroachment of human activity in natural spaces*,* and the release of pollutants can affect antimicrobial resistance*,* food safety and security*,* research and development of novel medicines*,* new emerging diseases associated with wildlife and domesticated species*,* increased pandemic risk*,* and overall health resilience* [26].*”* *“Invasive alien species (IAS)*,* one of the five direct drivers of global biodiversity loss*,* are species of plants*,* animals*,* and micro-organisms introduced to environments outside of their habitat of origin that*,* once established*,* threaten local biodiversity*,* ecosystems*,* and species*,* and the economy or society* [26].” *“Biodiversity is the variety of all living organisms on Earth. It includes terrestrial and aquatic animals*,* plants*,* fungi*,* and bacteria*,* and the genetic diversity within them* [26].*”*	Yes
South Africa’s 2nd National Biodiversity Strategy and Action Plan (2015–2025) [27]	Only fungi are mentioned: "*Species of plants, animals, birds, fish, frogs,reptiles, molluscs, insects and fungi are the building blocks of ecosystems* [27]	No
2024 USAID Biodiversity Policy* [13]	*“Ecosystem: A dynamic complex of plant*,* animal*,* fungi*,* and microorganism communities and their nonliving environment interacting as a functional unit* [13].*”*	Yes
Indonesian Biodiversity Strategy and Action Plan for 2015–2020 [28]	Discusses microbe diversity in detail. Examples below: *“Research of Indonesian microbial for agriculture (such as for producing natural herbicide*,* biological fertilizer*,* biological control for various types of pests and plant diseases)*,* for the health sector (among others as source of antibiotics*,* new bioactive compounds*,* ion-blocker for the treatment of diseases and virus infection antidote molecules*,* including avian influenza*,* etc.) and in the environmental sector for bioremediator*,* including to handle oil pollution*,* have been largely conducted by researchers in various research institutions*,* universities and private companies in Indonesia* [28].*”* *“Biodiversity is interpreted as all living creatures on earth*,* including all plant*,* animal and microbe species* [28].*”* *“Invasive microbes have the significant potential to change socioeconomic conditions through changes of the ecosystem diversity function*,* either the terrestrial as well as aquatic ecosystems. In general*,* invasive microbes are pathogenic to other organisms* [28].*”*	No
EU Biodiversity Strategy for 2030 [29]	None	No
African Union Biodiversity Strategy and Action Plan 2023–2030* [30]	None	No
Australia’s Strategy for Nature 2024–2030* [31]	*“Australia’s economy and future growth potential are inextricably linked to our natural resources (including plants*,* animals*,* water and even microbes)*,* many of which are finite or may be irreparable if managed unsustainably* [31].*”*	No

*Utilizes Kunming-Montreal Global Biodiversity Framework

## Barriers to inclusion of bacteria and AMR within biodiversity strategies

From our assessment, a critical question of whether environmental stakeholders actively consider bacteria or microbes a part of nature emerges. Certainly, bacteria are a vital part of the natural ecosystem as evidenced above. However, not all the documents referenced them as part of the ecosystem. Given that bacteria are an invisible part, it is easy for microbes and AMR to be forgotten, as it is much harder to track diversity loss in microbes compared to animals and plants. For instance, Australia’s Strategy for Nature states “*and even microbes*” when describing natural resources [31]. Indonesia, notably, spent a portion of its biodiversity strategy and action plan going into detail about microbe diversity [28]. Different locations might consider microbial diversity and communities as valuable resources over others, if they have unique and rich collections.

The goal of environmental strategies is often to provide recommendation and guidance to reach a specific target of or define a permissible level of pollution or emissions. However, in the context of antibiotic pollution (including agricultural runoff), is this possible? Is there any level that is permissible when it is known that even low levels of antibiotics pose a threat to AMR [32]. This speaks to the tension between antibiotics being pollutants [33] and essential tools [34], and to the difficulty in setting a safe exposure level for environmental antibiotic pollution when there are multiple drivers that influence clinical outcomes [35]. The 2024 UN Political Declaration on the High-level Meeting on Antimicrobial Resistance set a numerical target to reduce mortality associated with AMR by 10% by 2030 and a commitment to meaningfully reduce the quantity of antimicrobials globally in agri-food systems [36]. The absence of specific targets and laws for reduction of environmental antibiotic pollution hinders the use of regulatory instruments, which often rely on measurable objectives and benchmarks for compliance. For biodiversity, one market-based instrument are taxes on pesticides [37]. However, pesticides are known to be toxic to humans [37], while antibiotics are essential tools and medicines, so the immediate perceived threat and rationale differs.

A similar challenge is seen with biodiversity and climate change, in that while climate change and biodiversity crises are connected, they have largely been addressed independently [38]. Barriers include limited information on how approaches benefit each other and gaps in how to evaluate co-impacts, in addition to differences in funding. Furthermore, biodiversity and climate change agendas function under different levels of resources and political leverage, thus requiring higher levels of integration [38]. In general, remaining in silos (physical, organizational and experiential) is a common problem in public and private organizations and amongst policy makers which limits integration of agendas [39].

## Recommendations for integrating agendas

To bridge sectors, there needs to be clear connections on how policy actions are related, and the benefit of specific actions on each other. For biodiversity strategies, the exclusion of microbes from the GBF and regional and national strategies is a critical omission; we have outlined how AMR and microbes are linked to the goals of biodiversity (Table 1), which can be a starting point for addressing the limited integration in current documents (Table 2). Additionally, increased attention to biodiversity can be integrated into AMR policy documents, such as National Action Plans.

One current challenge, however, is consensus evidence on the relationship between AMR and biodiversity. Developing a monitoring framework or integrated surveillance that quantifies both reduction in antimicrobial use and AMR as well as loss of biodiversity can provide clear evidence that demonstrates relationships occurring in the real-world that policymakers may seek (as well as inform fundamental research). For example, antibiotic use and soil health on farms could be measured simultaneously (or data collated). Increased research (and funding for research) probing the relationship between environmental antibiotic use and biodiversity will help support evidence-based policy as well [40].

A central challenge to bridging silos and policy spaces is the difficulty in communicating and collaborating among different expertise, in which knowledges spaces may be unknown to each other [39]. By aligning goals and establishing accountability, stakeholders can establish better communication and connections across agendas [41]. Considering the initial step of aligning goals and agenda setting reveals different but related goals of AMR and biodiversity policies. AMR and indiscriminate antibiotic use can hinder many of the overarching goals of biodiversity strategies (e.g. sustainable agriculture, soil health). The overall end goal of AMR policy is to reduce AMR and antibiotic use, which can improve and support biodiversity goals. Thus, inclusion of reducing antibiotics and AMR can be seen as an important activity that supports the end goals of biodiversity.

## Key findings

Here, we examined the shared interests between antibiotic-reducing activities and biodiversity goals. We assessed the Kunming-Montreal Global Biodiversity Framework (GBF) and regional and national biodiversity strategies for inclusion of One Health AMR topics including antibiotics, antimicrobial resistance and recognition of microbes as a component of ecosystems and biodiversity. Overall, we found:

- While the GBF does not mention explicitly microbes or AMR, it contains multiple targets relevant to AMR and antibiotics.
- A review of seven biodiversity strategies from across the globe found limited mention of microbes, antibiotics, AMR or One Health.
- Areas where biodiversity strategies and AMR One Health strategies overlap include Pollution, Soil Health, Food Security, Ecosystem Functions and Sustainable Agriculture.
- Inclusion of activities to reduce antibiotic use and AMR contribute to biodiversity goals.

## Conclusions

Policies developed in isolation fail to consider cross-sectoral connections. One Health highlights the need to address interconnected health threats with collaboration between diverse stakeholders across disciplines, however it is unclear if these disciplines are brought into individual policies in practice. One Health AMR activities can have direct positive impacts on the goals of biodiversity policy, thus rationalizing their inclusion. Improved integrated monitoring for AMR, antibiotic use and biodiversity goals may help build the bridge between sectors.

## Data Availability

No datasets were generated or analysed during the current study.
